# Thromboembolic prevention and anticoagulant therapy during the COVID-19 pandemic: updated clinical guidance from the anticoagulation forum

**DOI:** 10.1007/s11239-022-02643-3

**Published:** 2022-05-17

**Authors:** Geoffrey D Barnes, Allison Burnett, Arthur Allen, Jack Ansell, Marilyn Blumenstein, Nathan P Clark, Mark Crowther, William E Dager, Steven B. Deitelzweig, Stacy Ellsworth, David Garcia, Scott Kaatz, Leslie Raffini, Anita Rajasekhar, Andrea Van Beek, Tracy Minichiello

**Affiliations:** 1 University of Michigan, 2800 Plymouth Rd, B14 G214, 48109-2800 Ann Arbor, MI USA; 2 University of New Mexico Health Sciences Center, Albuquerque, NM USA; 3 VA Salt Lake City Health Care System, Salt Lake City, UT USA; 4 Professor of Medicine, Hofstra/Northwell School of Medicine , New York, USA; 5 Children’s Hospital of Philadelphia , Philadelphia, PA, USA; 6 Kaiser Permanente Colorado, Colorado University Skaggs School of Pharmacy, Aurora, CO USA; 7 McMaster University, Hamilton, ON Canada; 8 UC Davis Medical Center, Sacramento, CA USA; 9 Ochsner Health System, New Orleans, LA USA; 10 Henry Ford Hospital, Detroit, MI USA; 11 University of Washington, Seattle, WA USA; 12 University of Pennsylvania Children’s Hospital of Philadelphia, Philadelphia, PA USA; 13 University of Florida Health, Visalia, CA USA; 14 Kaweah Health Medical Group/Visalia Medical Clinic, Visalia, CA USA; 15 University of California, San Francisco San Francisco VA Medical Center, San Francisco, CA USA

**Keywords:** Anticoagulation, COVID-19, Direct oral anticoagulant, Prophylaxis, Stewardship, Venous thromboembolism, Direct-acting oral anticoagulant, Low-molecular-weight heparin, Enoxaparin, rivaroxaban, Aspirin, Thrombosis and thrombocytopenia syndrome, Pregnancy, Thrombophilia

## Abstract

Thromboembolism is a common and deadly consequence of COVID-19 infection for hospitalized patients. Based on clinical evidence pre-dating the COVID-19 pandemic and early observational reports, expert consensus and guidance documents have strongly encouraged the use of prophylactic anticoagulation for patients hospitalized for COVID-19 infection. More recently, multiple clinical trials and larger observational studies have provided evidence for tailoring the approach to thromboprophylaxis for patients with COVID-19. This document provides updated guidance for the use of anticoagulant therapies in patients with COVID-19 from the Anticoagulation Forum, the leading North American organization of anticoagulation providers. We discuss ambulatory, in-hospital, and post-hospital thromboprophylaxis strategies as well as provide guidance for patients with thrombotic conditions who are considering COVID-19 vaccination.

**Supplementary Information:**

The online version contains supplementary material available at 10.1007/s11239-022-02643-3.

## Introduction and background

Early in the coronavirus disease 2019 (COVID-19) pandemic, thrombosis was identified as a key associated complication. Without the benefit of high-quality evidence, numerous expert guidance documents were published addressing the intensity of thromboprophylaxis in the hospital and non-hospital setting, the role of biomarkers to guide antithrombotic therapy, and best practices for minimizing COVID-19 exposure for patients on chronic anticoagulant therapy.[1–4] These documents were based largely on indirect evidence from non-COVID patients and relied on expert opinion. Since then, evidence in the prevention and treatment of COVID-19 has emerged. Clinical trials have addressed vaccine efficacy and safety, the role of steroids and the utility of numerous anti-viral therapies. Understanding of pathophysiologic mechanisms of thrombosis in COVID-19 has evolved, with recognition that patients may be at risk for both macrothrombotic events (e.g., deep venous thrombosis, pulmonary embolism) and immunothrombosis in situ. It has been proposed that anticoagulants may have pleiotropic antiviral and anti-inflammatory effects, in addition to thromboembolism prevention in hospitalized COVID-19 patients.[5–7] In light of the emerging evidence, we provide updated guidance for key areas of thromboembolism prevention and treatment for patients with COVID-19 (Table 1).

**Table 1:** Summary of Guidance Recommendations

Clinical Area	Recommendation
Adult patients	
Thromboembolic Prevention - Ambulatory	We **recommend** against any specific antithrombotic preventative therapy for ambulatory (non-hospitalized) adult patients with mild COVID-19 infection who have no other indication for antithrombotic therapy.
	We **recommend** patients on antithrombotic therapy prior to diagnosis of COVID-19 continue their antithrombotic therapy unless a significantly elevated risk of bleeding has developed
Thromboembolic Prevention – Hospitalized (all)	We **recommend** that all patients hospitalized with COVID-19 receive at least standard dose thromboprophylaxis.
	We *suggest* that non-heparin anticoagulants (i.e., direct oral anticoagulants) be avoided when therapeutic intensity thromboprophylaxis is utilized.
	We **recommend** that “intermediate” intensity thromboprophylaxis and/or antiplatelet agents only be used in the setting of a clinical trial for hospitalized patients with COVID-19.
	We *suggest* that adult patients admitted to the hospital for COVID-19 remain on the intensity of VTE thromboprophylaxis that was initiated at hospital admission as long as their bleeding risk is not significantly elevated.
	In patients admitted to the hospital for indications other than COVID-19 but incidentally found to have COVID-19 infection, we **recommend** standard dose thromboprophylaxis with LMWH or UFH unless specific contraindications exist.
	We *suggest* that the use of adjunctive therapies (i.e., statins, antiplatelets) only be used in the setting of a clinical trial.
Thromboembolic Prevention – Hospitalized (non-critically ill)	We *suggest* that clinicians consider the use of therapeutic intensity LMWH or UFH thromboprophylaxis for non-critically ill patients at increased risk of disease progression or thromboembolism and who are not high risk for anticoagulant-related bleeding (Table 2).
Thromboembolic Prevention – Hospitalized (critically ill)	We **recommend** that adult patients who are critically ill at the time of hospitalization receive standard dose thromboprophylaxis instead of intermediate- or therapeutic intensity thromboprophylaxis.
Thromboembolic Prevention – Post-hospital	We **recommend** that clinicians not routinely use post-hospital thromboprophylaxis after discharge following hospitalization for COVID 19 for all patients, including those who may have received therapeutic intensity anticoagulation for thromboprophylaxis.
	We *suggest* post-hospital thromboprophylaxis with rivaroxaban 10 mg daily for 35 days following a hospitalization for COVID-19 may be considered in select patients at increased risk of thromboembolism (e.g., IMPROVE VTE score ≥ 4 or score 2–3 with elevated D-dimer) and not at increased risk of bleeding regardless of the intensity of their inpatient thromboprophylaxis.
	We **recommend** clear documentation and communication of indication and intended duration of post-hospital thromboprophylaxis to providers and next care settings to avoid unnecessarily prolonged exposure to anticoagulation.
VTE Treatment	We **recommend** that most patients who are diagnosed with VTE while hospitalized for COVID-19 receive anticoagulation for a minimum of three to six months, in accordance with recent guidelines for VTE with a transient provoking risk factor.
	We *suggest* a finite course of anticoagulation (e.g., 3–6 months) rather than continuing anticoagulation long-term for secondary prevention in most patients with COVID-19 associated VTE. The duration should be a minimum of three months and defined by the presence or absence of persistent risk factors and the patients’ return to their baseline functional status.
COVID-19 Vaccination	We **recommend** that all patients with a history of thromboembolism, thrombophilia, or current use of anticoagulation be offered and encouraged to receive COVID-19 vaccination.
	We **recommend** that anticoagulation not be withheld for vaccine administration.
	If a patient currently uses anticoagulant therapy, we **recommend** that pressure be held at the site of vaccine administration for 5 min to minimize any risk of injection-related bleeding.
	We **recommend** that standard warfarin monitoring schedules not be altered in relation to vaccine administration for most patients. Individual patients experiencing significant symptoms, such as fever or dietary disruption, should contact their prescriber to determine if additional INR follow up is warranted.
Pediatric patients	
	We *suggest* that clinicians consider thromboprophylaxis with twice daily LMWH (e.g., enoxaparin 0.5 mg/kg BID) targeted to an anti-Xa activity level of 0.2 to < 0.5 IU/ml (in combination with mechanical prophylaxis when feasible) in pediatric patients hospitalized with acute COVID-19 or MIS-C and one or more additional risk factors associated with hospital-acquired VTE (e.g., central venous catheter, age > 12 years, immobility, mechanical ventilation, history of VTE, obesity, active malignancy, etc.) OR markedly elevated D-dimer, as long as the patient is not high risk for bleeding.
	We **recommend** against thromboprophylaxis in hospitalized children who are incidentally found to have asymptomatic SARS-CoV-2 in the absence of other VTE risk factors that would normally merit prophylaxis.
	Because of very limited published evidence, we *suggest* that post-discharge thromboprophylaxis be considered on a case-by-case basis in highly select pediatric patient with multiple ongoing risk factors.
Obstetric patients	
	We **recommend** against routine thromboprophylaxis for pregnant women found to be COVID-19 positive and not requiring admission to the hospital. Patients should be encouraged to stay hydrated and ambulate at home.
	For pregnant women requiring hospitalization for COVID-19, we **recommend** use of thromboprophylaxis in accordance with existing obstetric guidelines for non-COVID-19 positive women. Readers are referred to those population-specific resources for thromboprophylaxis dosing recommendations that take into consideration trimester, as well as ante- and post-partum needs.
	For pregnant women already receiving anticoagulant prophylaxis or treatment prior to hospital admission for COVID-19, we **recommend** continuing those therapies during admission and beyond if indicated.
	We *suggest* against routine post-discharge prophylaxis for COVID-19 positive obstetric patients unless they otherwise meet criteria for extended obstetric prophylaxis for non-COVID populations.
Other Special Populations	
Long-term Care Facilities	We *suggest* that patients who reside in long-term care settings who are ill enough with COVID-19 to be considered for hospital admission but remain in the long-term care facility be offered standard intensity thromboprophylaxis (Table 3) for up to 10–14 days only if this aligns with their goals of care.
Patients with Thrombophilia	We **recommend** that all adult patients with a known thrombophilia receive at least standard intensity thromboprophylaxis when hospitalized for COVID-19, unless already on chronic therapeutic-intensity anticoagulation for the thrombophilia.
Patients with anticoagulation use prior to hospitalization	We **recommend** that patients who are admitted to the hospital for COVID-19 infection be assessed for the use of ongoing outpatient anticoagulation.
	We **recommend** that the outpatient anticoagulation regimen be continued during the hospitalization for COVID-19 unless there are conditions that will preclude safe use (e.g., acute renal failure, anticipated invasive procedures, significant drug-drug interactions). Therapeutic or prophylactic UFH or LMWH may be substituted according to the clinical scenario. Dosing intensity of UFH or LMWH should take into consideration the underlying non-COVID indication for anticoagulation as well as COVID-related thromboprophylaxis needs as described in the above recommendations.
	We **recommend** that patients who were receiving reduced dose or very low dose anticoagulation (i.e., rivaroxaban 2.5 mg twice daily) prior to admission and who are hospitalized for COVID-19 substitute either receive either standard dose or therapeutic intensity thromboprophylaxis with LMWH or UFH as clinically appropriate (see recommendations above).

VTE – venous thromboembolism, ULN – upper limit of normal, ICU – intensive care unit, DOAC – direct oral anticoagulant, LMWH – low-molecular-weight heparin, UFH – unfractionated heparin

COVID-19 vaccination dramatically reduces the risk of severe infection, and therefore also greatly reduces the risk of infection-associated thrombosis. Patients with prior venous thromboembolism (VTE) may be leery of vaccination due to concerns that the immunization will increase the risk of thrombosis. Providers should educate patients about the thrombotic risk associated with COVID-19 infection and emphasize that COVID-19 itself is likely to greatly accentuate their risk of thromboembolism orders of magnitude greater than any potential thromboembolism risk following vaccination.[8] A past history of venous thromboembolism and enhanced risk for venous thromboemboli do not constitute a rationale for not being vaccinated.

## Methods

As with previous Anticoagulation Forum guidance documents, we prioritized a set of key questions and/or clinical practice areas relevant to thrombosis prevention and treatment among patients at risk for or diagnosed with COVID-19. These questions and clinical practice topics were selected through discussion and consensus among the authors as well as by members of the Anticoagulation Forum through an online survey (Table 1). We searched PubMed to identify evidence related to these questions. This search was supplemented by articles from the authors’ files and manual review of references. For each question or topic area, a summary of the evidence is provided, followed by guidance representing consensus of the authors.

Guidance statements with strong evidence-base or broad expert consensus are described using the term “recommend.” Statements with less strong evidence or less consensus are described using “suggest.” As with all clinical guidance, these statements should not replace clinical judgement by the treating care team and review of evolving clinical evidence.

## Thromboembolic Prevention – Adult Non-Pregnant Populations

### Ambulatory Patients with COVID-19

For ambulatory (non-hospitalized) adult patients with COVID-19, the overall risk of thromboembolism does not appear to be markedly elevated.[9, 10] The National Institutes of Health (NIH)-sponsored ACTIV-4b clinical trial comparing placebo, aspirin, and two doses of apixaban for patients with COVID-19 infection who did not require hospitalization was recently stopped due to a low number of thromboembolic events across all treatment groups.[11] Overall rates of death, symptomatic arterial or venous thromboembolism, myocardial infarction, stroke, or cardiopulmonary hospitalization were extremely low in all groups (0.7–1.4%) without any meaningful difference between groups.

For patients who already take chronic antithrombotic therapy (e.g., stroke prevention in atrial fibrillation, secondary prevention of coronary artery disease), observational studies have shown mixed results. Some have found an association between use of antithrombotic agents and improved outcomes while others have failed to demonstrate such a benefit.[12, 13] Nonetheless, even in the presence of COVID-19 infection, these patients usually retain their indication for antithrombotic therapy. Therefore, unless their clinical condition changes and the risk of bleeding significantly increases, continued use of the chronic antithrombotic agent is generally advised.

***Guidance Recommendation***:

1. We **recommend** against any specific antithrombotic preventative therapy for ambulatory (non-hospitalized) adult patients with mild COVID-19 infection who have no other indication for antithrombotic therapy.
2. We **recommend** patients on antithrombotic therapy prior to diagnosis of COVID-19 continue their antithrombotic therapy unless a significantly elevated risk of bleeding has developed..

### Non-Critically Ill Hospitalized Patients with COVID-19

For adult patients who are not critically ill but still hospitalized for COVID-19 and do not otherwise have an indication for anticoagulation, four completed clinical trials have explored the potential role of different intensities of anticoagulation (online appendix). In the multiplatform clinical trials (ATACC, ACTIV-4 A, REMAP-CAP) of more than 2200 patients, use of therapeutic-intensity heparin (primarily enoxaparin) was superior to ‘usual care’ pharmacologic thromboprophylaxis (71.7% standard dose, 26.5% intermediate dose) with heparin (primarily low-molecular-weight heparin [LMWH]) for increasing organ support-free days which is the number of days without cardiovascular (e.g. use of vasopressor or inotropic medications) or respiratory (e.g. use of high-flow nasal cannula, noninvasive or invasive mechanical ventilation) support (80.2% vs. 76.4%, adjusted difference 4.0%, 95% CI 0.5–7.2%).[14] Presence of elevated baseline D-dimer did not significantly alter the primary outcome. Overall survival until hospital discharge was not different among the two treatment arms (adjusted risk difference 1.3%, 95% CI − 1.1 to 3.2%), however there was a reduction in the secondary outcome of major thromboembolic events or death (2.6%, 95% CI 0.2–4.4%) favoring therapeutic heparin. There was a numerically higher risk of major bleeding with treatment-dose heparin (1.9% vs. 0.9%) that did not reach statistical significance. Of note, these trials screened more than 13,000 patients. Key strengths of this trial are the large size, global site inclusion, and use of blinded adjudication of event outcomes. But interpreting clinicians should be aware that > 12,000 of the screened patients were not included and that approximately one-quarter of patients received an intermediate-dose of heparin. This may have blunted any differences in efficacy and safety outcomes between the two treatment groups. Furthermore, interpretation of these absolute risk differences should be done carefully given the adaptive, Bayesian nature of the study design rather than traditional 1:1 randomization as is often seen in most traditional randomized clinical trials. Patients at high risk of bleeding were excluded from the study, which may partially explain the low rate of major bleeding in the trial population. The definition of ‘major’ thrombotic events did not include deep vein thrombosis; however, the addition of deep vein thrombosis did not alter the results of the secondary outcome analysis. There is also some question as to the value patients place on the outcome of the absolute number of organ support-free days versus any need for organ support, objective thromboembolic events, or overall survival. Finally, while large and well conducted, there are concerns about the relatively low rate of symptomatic thromboembolic events and lack of mortality benefit.

In the RAPID COVID COAG study, 465 moderately ill adult patients admitted with COVID-19 and elevated D-dimer levels were randomized to receive therapeutic intensity or standard dose prophylactic heparin.[15] Greater than 90% of both treatment arms received LMWH (vs. unfractionated heparin). Patients were included if their D-dimer level was > 2-times the hospital upper limit of normal (ULN) or if it was > 1-time the upper limit of normal along with documented hypoxia. The primary endpoint of death, invasive or non-invasive mechanical ventilation, or ICU admission was not statistically different between the two groups (16.2% versus 21.9% for therapeutic and standard intensity prophylaxis, respectively, OR 0.69, 95% CI 0.43–1.10). Interestingly, the secondary outcome of all-cause mortality was reduced with therapeutic intensity prophylaxis (1.8% vs. 7.6%, OR 0.22, 95% CI 0.07–0.65) while the VTE risk was low and similar in the two groups (0.9% vs. 2.5% in the therapeutic and prophylactic groups, respectively, OR 0.34, 95% CI 0.07–1.71). The rate of major bleeding was similar in both groups (0.9% vs. 1.7%, OR 0.52, 95% CI 0.09–2.85). VTE risk was low and similar in the two groups − 0.9% versus 2.5% in the therapeutic and prophylactic groups, respectively (OR 0.34, 95% CI 0.07–1.71). This trial screened nearly 4000 patients to enroll and randomize 465 patients. Strengths of this trial include the use of blinded adjudication of outcomes and a set of study endpoints that are patient centric. However, RAPID COVID COAG was likely underpowered to detect a difference in key outcome measures.

In the HEP-COVID trial, 257 patients admitted to the hospital with COVID-19 and D-dimer levels > 4-times the ULN were randomized to receive standard dose or therapeutic intensity thromboprophylaxis with heparin (mostly LMWH).[16] Patients were stratified at the time of randomization into ICU (32.8%) and non-ICU (67.2%) cohorts. Use of therapeutic intensity thromboprophylaxis reduced the primary efficacy outcome (arterial or venous thromboembolism or death) significantly (28.7% versus 41.9%, RR 0.68, 95% CI 0.49–0.96), which was driven by the non-ICU cohort (16.7% versus 36.1%, RR 0.46, 95% CI 0.27–0.81). Major bleeding was non-significantly elevated in patients receiving therapeutic intensity thromboprophylaxis (4.7% versus 1.6%, RR 2.88, 95% CI 0.59–14.02) overall but rates were similar among the non-ICU stratum (2.4% versus 2.3%). Of note, this trial screened more than 11,000 patients to enroll 257, largely excluding patients who did not meet criteria or lacked sufficiently elevated D-dimer levels, which raises concerns about external validity. Furthermore, this study relied on local adjudication of clinical events, which can lead to biased assessments when treatment allocation is known. The analysis also included asymptomatic VTE events identified on routine lower extremity compression ultrasound study at hospital day 10 ± 4 (or hospital discharge), the clinical importance of which are unknown. However, symptomatic DVT was significantly reduced (5.4% vs. 15.3%, RR 0.35, 95% CI 0.15–0.81) while symptomatic VTE was non-significantly reduced (1.6% vs. 2.4%, RR 0.38, 95% CI 0.12–1.19).

In the ACTION trial, 615 patients randomized to treatment-dose rivaroxaban experienced similar risk of death, duration of hospitalization, and duration of supplemental oxygen as compared to prophylactic dose heparin.[17] The composite thrombotic outcome (VTE, myocardial infarction, stroke, and major adverse limb events) was similar in the two treatment groups (7% vs. 10%, RR 0.75, 95% CI 0.45–1.26). The outcome of ISTH-defined major bleeding was higher in the rivaroxaban group than with prophylactic dose heparin (8% vs. 2%, RR 3.64, 95% CI 1.61–8.27).

A few key points can be abstracted from these four published trials. First, use of therapeutic intensity non-heparin thromboprophylaxis (e.g., rivaroxaban) did not demonstrate benefit over standard dose thromboprophylaxis with heparin and increased the risk of major bleeding. Second, each of the randomized trials used a different D-dimer threshold for inclusion and/or analysis subgroups. Third, each of these trials had strict inclusion and exclusion criteria that selected for low bleeding risk patients, like most anticoagulant trials for a new indication, suggesting that therapeutic intensity anticoagulation, if used, should be applied only after weighing risks and benefits.

#### Guidance Recommendation:

3) We **recommend** that all patients hospitalized with COVID-19 receive at least standard dose thromboprophylaxis.
4) In patients admitted to the hospital for indications other than COVID-19 but incidentally found to have COVID-19 infection, we **recommend** standard dose thromboprophylaxis with LMWH or unfractionated heparin (UFH) unless specific contraindications exist.
5) We *suggest* that clinicians consider the use of therapeutic intensity LMWH or UFH thromboprophylaxis for non-critically ill patients at increased risk of disease progression or thromboembolism and who are not high risk for anticoagulant-related bleeding (Table 2).
6) We **recommend** that “intermediate” intensity thromboprophylaxis and/or antiplatelet agents only be used in the setting of a clinical trial for hospitalized patients with COVID-19.

**Table 2:** Suggested Criteria for Therapeutic Intensity Thromboprophylaxis for Moderately Ill Patients with COVID-19

Criteria for Potential Disease Progression	Bleeding Risk Factors That Must Not Be Present
Admitted for COVID-19 infection (not an incidental finding)	End stage renal disease on dialysis
Supplemental oxygen requirement	Advanced liver disease or cirrhosis
Elevated d-dimer (> 2–4 times ULN)	Severe thrombocytopenia
	Use of dual antiplatelet therapy
	Need for therapeutic anticoagulation (e.g., atrial fibrillation, mechanical heart valve)
	Severe anemia
	Contraindication to heparin agents or Heparin-inducted thrombocytopenia
	Recent bleeding
	Bleeding disorder

To consider therapeutic intensity thromboprophylaxis for moderately ill patients with COVID-19, they should meet all 3 criteria for disease progression as well as not having any bleeding risk factors. Note that the risk factors listed are not exhaustive

ULN – upper limit of normal

Given that there are no head-to-head comparisons of various heparin agents, use of either LMWH or UFH can be considered for thromboprophylaxis. However, most heparin used in the clinical trials was LMWH which also affords less exposure and personal protective equipment utilization.

Suggested UFH and LMWH regimens for hospitalized non-pregnant adults, including adjustments for renal impairment and obesity, are reflected in Table 3.

**Table 3:** Dosing of COVID-19 Thromboprophylaxis in Hospitalized, Non-Pregnant Adults

Category	Enoxaparin	UFH	Dalteparin	Rivaroxaban
**Standard-intensity**	40 mg SQ daily	5000 units SQ BID-TID	5000 units SQ daily	N/A
*Renal impairment*				
CrCl 20–30 ml/min	30 mg SQ daily	5000 units SQ BID-TID	Usual dose with caution *or* use UFH	N/A
CrCl < 20 ml/min[41]	Use UFH		Use UFH	
*Obesity*				
BMI > 40 kg/m^2^	40 mg SQ BID[42] *or* 0.5 mg/kg SQ daily[43]	7500 units SQ BID-TID[44]	7500 units SQ daily[45]	N/A
*Renal impairment + obesity*	Use UFH	7500 units SQ BID-TID[44]	Use UFH	
**Therapeutic-intensity**	1 mg/kg SQ BID	Per local IV protocol	100 units/kg SQ BID	N/A
*Renal impairment*				
CrCl 20–30 ml/min	1 mg/kg SQ daily	Per local IV protocol	Usual dose with caution *or* use IV UFH per local protocol	N/A
CrCl < 20 ml/min	Use IV UFH per local protocol		Use IV UFH per local protocol	
*Obesity*				
BMI > 40 kg/m2	N/A (weight-based)	Per local IV protocol	N/A (weight-based)	N/A
*Renal impairment + obesity*	Use IV UFH per local protocol	Per local IV protocol	Use IV UFH per local protocol	N/A
**Extended duration**	N/A	N/A	N/A	10 mg PO daily x 35–39 days[46, 47]
*Renal impairment*				
CrCl < 30 ml/min	N/A	N/A	N/A	Avoid use^#^
*Obesity*				
	N/A	N/A	N/A	10 mg PO daily x 35–39 days

*Dosing list is not exhaustive, but represents the most commonly used regimens in the cited COVID-19 clinical trials and in routine clinical practice

#Rivaroxaban is FDA-approved for this indication in patients with CrCl ≥ 15 ml/min, but based on minimal evidence. Utilize with caution in this population

Mg – milligrams, SQ – subcutaneous, BID – twice daily, TID – three times daily, UFH – unfractionated heparin, CrCl – creatinine clearance, IV – intravenous, PO - oral

Whenever therapeutic intensity of UFH, LMWH, or other anticoagulants are given for thromboprophylaxis and not for treatment of presumed or confirmed thromboembolism, this should be clearly documented in the patient chart.

### Critically Ill Hospitalized Patients with COVID-19

Three studies have examined the use of non-standard doses of heparin for thromboprophylaxis in critically ill adult patients with COVID-19 (online appendix). In the multi-platform trials, patients admitted to the ICU and requiring organ support (e.g., high-flow oxygen, invasive ventilation, vasopressor or inotropic support) did not experience benefit when receiving therapeutic intensity thromboprophylaxis (primarily LMWH) as compared to ‘usual care’ thromboprophylaxis (primarily LMWH).[18] Of note, use of “intermediate” intensity thromboprophylaxis was quite common (51.7%) in the ‘usual care’ thromboprophylaxis group. Two trials have evaluated intermediate versus standard dose thromboprophylaxis in this population and failed to find a statistically significant benefit. In the INSPIRATION trial, patients admitted to the ICU did not experience benefit when receiving intermediate-dose enoxaparin (1 mg/kg daily) as compared to standard dose thromboprophylactic enoxaparin (40 mg daily).[19] Finally, a small, multi-center randomized trial of 176 patients with COVID-19 admitted to the ICU found a non-significant reduction in 30-day all-cause mortality associated with intermediate-dose enoxaparin as compared to standard dose thromboprophylactic enoxaparin (15% vs. 21%, OR 0.66, 95% CI 0.30–1.45) with a low rate of major bleeding (2% in each arm).[20].

Of note, approximately one-third of patients in the previously described HEP-COVID study required nonrebreather oxygen mask or more intensive respiratory support.[16] The primary efficacy endpoint (a composite of arterial thromboembolism, VTE, or death) was not significantly reduced in the stratum of patients admitted to the ICU (51.1% vs. 55.3%, RR 0.92, 95% CI 0.62–1.39).

#### *Guidance Recommendation*:

7) We **recommend** that adult patients who are critically ill at the time of hospitalization receive standard dose thromboprophylaxis instead of intermediate- or therapeutic intensity thromboprophylaxis.

Suggested dosing regimens for hospitalized non-pregnant adults, including adjustments for renal impairment and obesity, are reflected in Table 3.

### Antiplatelet Use

Two trials have explored the use of antiplatelet therapy in patients hospitalized with COVID-19. The RECOVERY trial enrolled adult patients in the United Kingdom, Indonesia, and Nepal who were hospitalized with COVID-19.[21] Patients were randomized to receive aspirin 150 mg daily or usual care. Among the 14,892 patients who were randomized, the rate of 28-day all-cause mortality was similar in the two groups (17% vs. 17%, RR 0.96, 95% CI 0.89–1.04). There was also no significant difference in the median time to discharge alive (8 vs. 9 days). In the ACTIVE-4a study, 562 non-critically ill patients hospitalized for COVID-19 in Brazil, Italy, Spain, and the United States were randomized to receive therapeutic intensity heparin plus a P2Y12 inhibitor (ticagrelor preferred) or therapeutic intensity heparin alone.[22] The primary outcome of organ support-free days was similar in both treatment groups (21 vs. 21, adjusted OR 0.83, 95% CI 0.55–1.25). There was also no difference in the rate of survival to hospital discharge or major bleeding.

#### Guidance Recommendation:

8) *We* ***recommend*** *that patients hospitalized with COVID-19 do not receive antiplatelet therapy (e.g., aspirin, P2Y12 inhibitor) for the specific purpose of preventing thromboembolism or COVID-19 disease progression.*

### Hospital Transitions of Care

Many patients who are hospitalized for COVID-19 infection will require transfers of care between the floor and ICU and vice versa. No studies have specifically compared different anticoagulation strategies when patients initially admitted to one unit (e.g., floor) require a chance in level of care (e.g., transfer to an ICU). The study protocols of the various randomized trials generally recommended patients remain on their initial intensity of anticoagulation even when transferring between different levels of care (e.g., floor to ICU) for up to 14 days or until recovery since the patients were enrolled and randomized shortly after hospital admission.[14, 18] Any decisions to use higher intensity anticoagulation should be clearly documented to provide guidance on approaches to post ICU management. Furthermore, there is no prospective clinical evidence to support the use of serial D-dimer testing to guide the intensity of antithrombotic therapy.

#### Guidance Recommendation:

9) We *suggest* that adult patients admitted to the hospital for COVID-19 remain on the intensity of VTE thromboprophylaxis that was initiated at hospital admission as long as their bleeding risk is not significantly elevated.

Thus, a moderately-ill patient with COVID-19 admitted to the ward and started on therapeutic intensity thromboprophylaxis should continue therapeutic intensity thromboprophylaxis when transferring to the ICU. The dose should be reduced to standard dose thromboprophylaxis when clinically necessary based on bleeding risk.

Similarly, patients initially admitted to the ICU for organ support and started on standard dose thromboprophylaxis should continue standard dose thromboprophylaxis when they transfer to the floor.

Patients should receive therapeutic intensity anticoagulation if a thromboembolic event is confirmed or highly suspected, and bleeding risk is not prohibitively high.

### Post-hospital Period

Observational studies have reported conflicting results regarding post-hospital VTE risk. In most reports, the observed risk of VTE was similar to patients without COVID-19.[9, 23–25] However, in one report, the risk of post-hospital VTE was elevated compared to patients without COVID-19.[26] Most recently, the MICHELLE trial randomized 320 patients hospitalized for COVID-19 who were receiving standard dose thromboprophylaxis during their admission and were considered at increased risk for post-discharge events, with an IMPROVE VTE score of ≥ 4 or an IMPROVE VTE score of 2–3 plus a D-dimer > 500 ng/ml at discharge.[27] Patients at increased risk of bleeding, such as those with bleeding in prior 3 months, on dual antiplatelet therapy or with chronic kidney disease, were excluded. Only 11 patients with creatinine clearance 30–50 ml/min were included in the study. Enrolled subjects were randomized to rivaroxaban 10 mg by mouth daily for 35 days or no post-discharge thromboprophylaxis. Post-hospital thromboprophylaxis with rivaroxaban reduced the primary composite outcome of symptomatic venous or arterial thromboembolism, VTE-related death, bilateral VTE, myocardial infarction, non-hemorrhagic stroke, major adverse limb event or cardiovascular death compared to no intervention (3.14% VS. 9.43%, RR 0.33, 95% CI 0.13–0.90). There were no major bleeds in either group. However, it should be noted that the MICHELLE trial screened 997 patients in order to enroll 320, suggesting that post-hospital extended thromboprophylaxis is not appropriate for all patients hospitalized with COVID-19. Note that the IMPROVE VTE score used in the MICHELLE trial gave 1 point for immobilization (confined to bed or chair with or without bathroom privileges) that lasted ≥ 1 day. This contrasts with the original IMPROVE VTE score which defined immobilization as ≥ 7 days immediately prior to and including hospitalization.[28].

#### Guidance Recommendation:

10) We **recommend** that clinicians not routinely use post-hospital thromboprophylaxis after discharge following hospitalization for COVID 19 for all patients, including those who may have received therapeutic intensity anticoagulation for thromboprophylaxis.
11) We *suggest* post-hospital thromboprophylaxis with rivaroxaban 10 mg daily for 35 days following a hospitalization for COVID-19 may be considered in select patients at increased risk of thromboembolism (e.g., IMPROVE VTE score ≥ 4 or score 2–3 with elevated D-dimer at hospital discharge) and not at increased risk of bleeding regardless of the intensity of their inpatient thromboprophylaxis.
12) We **recommend** clear documentation and communication of indication and intended duration of post-hospital thromboprophylaxis to providers and next care settings to avoid unnecessarily prolonged exposure to anticoagulation.

## Thromboprophylaxis for Pediatric Patients with COVID-19

In general, children with SARS-CoV2 have much milder infections compared to adults, rarely requiring hospitalization. However, more severe acute COVID-19, characterized by the typical respiratory phenotype, does occur and is more common in children with underlying medical conditions. Children may also develop a post-infectious multisystem inflammatory syndrome (MIS-C) that manifests with fever, cardiovascular shock, hyperinflammation and multi-system involvement. Rates of thrombotic complications in children with COVID-19 and MIS-C have not been well established, but a multi-center retrospective cohort study reported radiologically confirmed venous or arterial thrombosis in 2.1% (9/426) and 6.5% (9/138) of children hospitalized with acute COVID-19 and MIS-C respectively. Risk factors for thrombosis included age > 12 years, cancer, presence of a central venous catheter and MIS-C.[29] D-dimer > 5x upper limit of normal was also a risk factor in the univariate model. The mortality rate in children hospitalized with COVID-19 or MIS-C was 2.3%, but was 28% in those with thrombosis, suggesting thrombosis may contribute to mortality. Similar to what has been reported in adult studies, a significant proportion of VTE (71%) occurred in children who were already receiving anticoagulant prophylaxis. There was wide variation in the intensity of anticoagulation used for thromboprophylaxis among centers; no patient developed a post-discharge VTE.[30].

Randomized trials evaluating the efficacy and intensity of anticoagulant prophylaxis in COVID-19 pediatric patients have not yet been conducted, although prophylaxis is frequently used in tertiary care centers. In the interim, we suggest adhering to the Pediatric/Neonatal Hemostasis and Thrombosis Scientific Subcommittee of the International Society on Thrombosis and Hemostasis expert-consensus- based guidance for thromboprophylaxis in this population.[31].

### Guidance recommendations:

13) We *suggest* that clinicians consider thromboprophylaxis with twice daily LMWH (e.g., enoxaparin 0.5 mg/kg BID) targeted to an anti-Xa activity level of 0.2 to < 0.5 IU/ml (in combination with mechanical prophylaxis when feasible) in pediatric patients hospitalized with acute COVID-19 or MIS-C and one or more additional risk factors associated with hospital-acquired VTE (e.g., central venous catheter, age > 12 years, immobility, mechanical ventilation, history of VTE, obesity, active malignancy, etc.) OR markedly elevated D-dimer, as long as the patient is not high risk for bleeding.
14) We **recommend** against thromboprophylaxis in hospitalized children who are incidentally found to have asymptomatic SARS-CoV-2 in the absence of other VTE risk factors that would normally merit prophylaxis.
15) Because of very limited published evidence, we *suggest* that post-discharge thromboprophylaxis be considered on a case-by-case basis only in highly select pediatric patients with multiple ongoing risk factors.

The expert panel acknowledged the lack of pediatric data to support these recommendations, particularly with regard to intensity of anticoagulation, and called for additional investigation in this area.

## Thromboprophylaxis in Obstetric Patients with COVID-19

Randomized trials of thromboprophylaxis in adult COVID-19 patients have excluded pregnant women. Thus, there is no high-quality evidence to inform best approaches in COVID-19 positive obstetric patients. Pregnancy itself is a hypercoagulable condition and paired with acute illness, reduced mobility and dehydration may place pregnant women at significant risk of VTE. Retrospective analyses have reported thromboembolic complications in pregnant women with COVID-19, but it is unknown if the incidence is higher than in non-pregnant women. Routine use of thromboprophylaxis for pregnant women who are COVID-19 positive but do not require hospitalization is not required.[32] However, thromboprophylaxis should be considered for hospitalized pregnancy according to existing population-specific societal guidelines, taking into consideration patient characteristics (e.g., renal function, obesity) and obstetrical status (e.g., trimester, timing relative to expected labor and delivery). Additionally, close collaboration with obstetric and anesthesiology colleagues is recommended in the event of spontaneous delivery and/or need for epidural anesthesia during hospitalization. For pregnant women already receiving prophylactic or therapeutic anticoagulation who are admitted to the hospital for COVID-19, these therapies should be continued during the admission and for the appropriate duration after discharge. DOACs should be avoided in pregnancy as they have not been adequately studied and may cause fetal harm. There is no data to suggest need for routine thromboprophylaxis beyond hospitalization for COVID-19, and use should be limited to pregnant women who otherwise meet non-COVID-19 criteria for extended obstetric prophylaxis as per societal guidelines.[33].

### Guidance recommendations:

16) We **recommend** against routine thromboprophylaxis for pregnant women found to be COVID-19 positive and not requiring admission to the hospital. Patients should be encouraged to stay hydrated and ambulate at home.
17) For pregnant women requiring hospitalization for COVID-19, we **recommend** use of thromboprophylaxis in accordance with existing obstetric guidelines for non-COVID-19 positive women.[33–36] Readers are referred to those population-specific resources for thromboprophylaxis dosing recommendations that take into consideration trimester, as well as ante- and post-partum needs.
18) For pregnant women already receiving anticoagulant prophylaxis or treatment prior to hospital admission for COVID-19, we **recommend** continuing those therapies during admission and beyond if indicated.
19) We *suggest* against routine post-discharge prophylaxis for COVID-19 positive obstetric patients unless they otherwise meet criteria for extended obstetric prophylaxis for non-COVID populations.

**Thromboprophylaxis for Moderately Ill Patients in Long-term Care Facilities Who Are Not Transferred to the Hospital**.

Many patients who reside in long-term care facilities have multiple comorbidities and advanced age which presents increased risk for thrombotic complications during an acute, infectious illness such as COVID-19. When aligned with a patient’s goals of care, similar approaches to thromboprophylaxis can be used in the long-term care setting as would be used in the acute hospital setting if the patients is otherwise ill enough to warrant hospital admission.

#### Guidance recommendation:

20) We *suggest* that patients who reside in long-term care settings who are ill enough with COVID-19 to be considered for hospital admission but remain in the long-term care facility be offered standard intensity thromboprophylaxis (Table 3) for up to 10–14 days only if this aligns with their goals of care.

## Thromboprophylaxis in Patients with Known Thrombophilia

Patients with known thrombophilia are at increased risk for VTE from any acute medical hospitalization. However, data regarding any differential risk of thromboembolism while hospitalized with COVID-19 is lacking.

### Guidance Recommendation:

21) We **recommend** that all adult patients with a known thrombophilia receive at least standard intensity thromboprophylaxis when hospitalized for COVID-19, unless already on chronic therapeutic-intensity anticoagulation for the thrombophilia.

The presence of a known thrombophilia (and in the absence of chronic therapeutic-intensity anticoagulation for the thrombophilia) may sway the risk-benefit balance toward the use of therapeutic-intensity thromboprophylaxis in adult hospitalized medical patients with moderate COVID-19 illness as long as the risk of bleeding is not prohibitively high. Patients with a known thrombophilia who are admitted to the hospital with critically ill COVID-19 should typically be treated with standard thromboprophylaxis in a similar fashion to critically ill patients without thrombophilia.

Patients with a known thrombophilia that are not otherwise on chronic therapeutic-intensity anticoagulation may be candidates for post-hospital thromboprophylaxis if they meet the inclusion criteria stated above.

## Management of Patients on Anticoagulation Prior to Admission for COVID-19

Patients who use anticoagulation for chronic thromboembolic conditions (e.g., atrial fibrillation, VTE, mechanical valve) continue to be at risk of their baseline thromboembolic condition during their hospitalization for COVID-19. However, during hospitalization, they may also develop transient or long-standing bleeding risk factors (e.g., acute renal failure, need for invasive procedures, drug-drug interactions) that require adjustment to the anticoagulation treatment strategy.

### Guidance recommendation:

22) We **recommend** that patients who are admitted to the hospital for COVID-19 infection be assessed for the use of ongoing outpatient anticoagulation.
23) We **recommend** that the outpatient anticoagulation regimen be continued during the hospitalization for COVID-19 unless there are conditions that will preclude safe use (e.g., acute renal failure, anticipated invasive procedures, significant drug-drug interactions). Therapeutic or prophylactic UFH or LMWH may be substituted according to the clinical scenario. Dosing intensity of UFH or LMWH should take into consideration the underlying non-COVID indication for anticoagulation as well as COVID-related thromboprophylaxis needs as described in the above recommendations.
24) We **recommend** that patients who were receiving reduced dose or very low dose anticoagulation (i.e., rivaroxaban 2.5 mg twice daily) prior to admission and who are hospitalized for COVID-19 receive either standard dose or therapeutic intensity thromboprophylaxis with LMWH or UFH as clinically appropriate (see recommendations above).

## VTE Treatment

VTE guidelines favor a finite duration of anticoagulation therapy (e.g., 3–6 months) for VTE provoked by non-surgical transient risk factors such as medical illness, while also favoring ongoing therapy in the setting of significant persistent risk factors.[37, 38] Whether the risk of VTE recurrence among hospitalized patients with COVID-19 who develop VTE differs significantly from that of VTE provoked by other medical illness is not well established. Experience would suggest that the number of patients experiencing continued debility beyond three months is not insignificant. As such, while a finite duration of anticoagulation therapy should be the goal, having a low threshold for continuing therapy and reassessing patients’ recovery at regular intervals is a reasonable approach. Recurrence risk is likely low once the COVID-19 infection resolves and patients return to their baseline level of health.[39] Initial therapy likely will include LMWH or UFH, but can transition to DOAC therapy once the patient stabilizes prior to hospital discharge.

### Guidance Recommendation:

25) We **recommend** that most patients who are diagnosed with VTE while hospitalized for COVID-19 receive anticoagulation for a minimum of three to six months, in accordance with recent guidelines for VTE with a transient provoking risk factor.[37, 38].
26) We *suggest* a finite course of anticoagulation (e.g., 3–6 months) rather than continuing anticoagulation long-term for secondary prevention in most patients with COVID-19 associated VTE. The duration should be a minimum of three months and defined by the presence or absence of persistent risk factors and the patients’ return to their baseline functional status.

## Vaccination for patients on anticoagulation or prior VTE/thrombophilia

Vaccination is the leading strategy to control the COVID-19 pandemic. With significant protection against both infection and severe illness, major public health organizations strongly recommend that all eligible people are vaccinated against COVID-19. Given rare reports of both typical and unusual site thromboembolism following COVID-19 vaccination, many have wondered about the safety of COVID-19 vaccination for patients with a history of thromboembolism, thrombophilia, or current use of anticoagulation. Given the strong association between COVID-19 infection and thromboembolism, the rare occurrence of usual or unusual site thrombosis following COVID-19 vaccination is greatly outweighed by the benefit of preventing COVID-19 infection.[8] Furthermore, the occurrence of unusual site thromboembolism associated with thrombocytopenia (known as Vaccine-induced Thrombosis and Thrombocytopenia [VITT] or the Thrombosis and Thrombocytopenia Syndrome [TTS]) is driven by an immune-mediate process that is not linked to a history of thrombosis, thrombophilia, or current anticoagulant use.[40] A prior history of VTE or current use of anticoagulation should not influence the selection of one type of COVID-19 vaccine over another. There is no role for specific thromboprophylaxis (anticoagulation or antiplatelet) prior to receiving the COVID-19 vaccine.

### **Guidance Recommendation**:

27) We **recommend** that all patients with a history of thromboembolism, thrombophilia, or current use of anticoagulation receive COVID-19 vaccination if eligible.
28) We **recommend** that anticoagulation not be withheld for vaccine administration.
29) If a patient currently uses anticoagulant therapy, we **recommend** that pressure be held at the site of vaccine administration for 5 min to minimize any risk of injection-related bleeding.
30) We **recommend** that standard warfarin monitoring schedules not be altered in relation to vaccine administration for most patients. Individual patients experiencing significant symptoms, such as fever or dietary disruption, should contact their prescriber to determine if additional INR follow up is warranted.

Guidance recommendations for patients with thrombophilia, those in long-term care facilities, those using anticoagulation prior to COVID-19 hospitalization, and those with prior VTE considering COVID-19 vaccination are based on expert consensus given the lack of prospective clinical trial data or high-quality observational data.

## Conclusions

While the guidance statements above are based on the best available evidence at the current time, there are multiple trials underway that may refine the recommended approach to thromboprophylaxis in COVID-19. Clinicians are encouraged to use the rapidly emerging evidence in concert with expert guidance to make clinical management decisions for individual patients. Online resources, such as the Anticoagulation Forum Centers of Excellence, receive timely updates to provide clinicians reliable resources as new data emerge.

## Electronic Supplementary Material

Below is the link to the electronic supplementary material.

Supplementary Material 1
