# Supplementary material for: Thromboembolic prevention and anticoagulant therapy during the COVID-19 pandemic: updated clinical guidance from the anticoagulation forum
Source: J Thromb Thrombolysis. 2022 May 17;54(2):197–210. doi: 10.1007/s11239-022-02643-3 (PMC9111941; doi:10.1007/s11239-022-02643-3)
Supplement: Supplementary file 1 — Supplementary Material 1 [file 11239_2022_2643_MOESM1_ESM.docx]

**Online Table 1 – Clinical Trials in Non-critically Ill Patients**

| **Trial Name** | **Comparator Arms** | **Sample Size** | **Mean or Median BMI** | **Dosing adjustment for obesity** | **Percent with CKD** | **Dosing adjustment for CKD** | **Exclusion for CKD** |
| --- | --- | --- | --- | --- | --- | --- | --- |
| Multi-platform Trial | Therapeutic intensity heparin | 1190 | 29.8  (IQR 26.3-34.7) | No | 7.1 | Yes | No |
|  | Usual care with standard dose thromboprophylaxis | 1054 | 30.3  (IQR 26.7-34.9) | Yes | 6.7 | Yes |  |
| RAPID COVID COAG | Therapeutic intensity heparin | 228 | 30.3  (SD 6.4) | No (measure anti-Xa if BMI>40) | 20 | No | No |
|  | Standard dose thromboprophylaxis | 237 | 30.2  (SD 7.0) | Yes | 13 | No |  |
| HEP-COVID | Therapeutic intensity heparin | 129 | 31.2  (SD 9.3) | No | 3.9 | Yes | No |
|  | Standard dose thromboprophylaxis | 124 | 29.8  (SD 13.6) | No | 3.2 | Yes |  |
| ACTION | Treatment-dose rivaroxaban | 310 | 30.3  (SD 6.0) | No | 1.9 | Yes | Yes (CrCl<30 ml/min) |
|  | Standard dose thromboprophylaxis | 304 | 30.3  (SD 6.1) | No | 1.0 | Yes |  |

BMI – body mass index; CKD – chronic kidney disease; IQR – inter-quartile range; SD – standard deviation; CrCl – creatinine clearance

**Online Table 2 – Clinical Trials in Critically Ill Patients**

| **Trial Name** | **Comparator Arms** | **Sample Size** | **Mean or Median BMI** | **Dosing adjustment for obesity** | **Percent with CKD** | **Dosing adjustment for CKD** | **Exclusion for CKD** |
| --- | --- | --- | --- | --- | --- | --- | --- |
| Multi-platform Trial | Therapeutic intensity heparin | 534 | 30.4  (IQR 26.9-36.1) | No | 11.4 | Yes | No |
|  | Usual care with standard dose thromboprophylaxis | 564 | 30.2  (IQR 26.4-34.9) | Yes | 8.3 | Yes |  |
| INSPIRATION | Intermediate-dose enoxaparin | 276 | 26.7  (IQR 24.4-29.1) | Yes | Median Cr 1.1 mg/dL (IQR 0.9-1.2) | Yes | Yes |
|  | Standard dose thromboprophylaxis | 286 | 27.2  (IQR 24.3-29.1) | Yes | Median Cr 1.1 mg/dL (IQR 0.9-1.3) | Yes |  |
| Perepu et al | Intermediate dose enoxaparin | 87 | 30.0  (IQR 24.7-36.6) | Yes | n/a | No | Yes |
|  | Standard dose thromboprophylaxis | 86 | 30.7  (IQR 27.2-35.8) | Yes | n/a | No |  |

BMI – body mass index; CKD – chronic kidney disease; IQR – inter-quartile range; SD – standard deviation; Cr – serum creatinine
